# Impact of Emission Standards on Fine Particulate Matter Toxicity: A Long-Term Analysis in Los Angeles

**DOI:** 10.3390/toxics13020140

**Published:** 2025-02-18

**Authors:** Mohammad Mahdi Badami, Yashar Aghaei, Constantinos Sioutas

**Affiliations:** Department of Civil and Environmental Engineering, University of Southern California, Los Angeles, CA 90089, USA; mbadami@usc.edu (M.M.B.); yaghaei@usc.edu (Y.A.)

**Keywords:** non-tailpipe emissions, aerosol, metals and trace elements, DTT assay, air pollution regulations

## Abstract

This study examines long-term trends in fine particulate matter (PM_2.5_) composition and oxidative potential in Los Angeles based on data from the University of Southern California’s Particle Instrumentation Unit, with chemical composition retrieved from the EPA’s Air Quality System (AQS). While regulatory interventions have reduced PM_2.5_ mass concentration and primary combustion-related components, our findings reveal a more complex toxicity pattern. From 2001 to 2008, the PM_2.5_ oxidative potential, measured via the dithiothreitol (DTT) assay, declined from ~0.84 to ~0.16 nmol/min/m^3^ under stringent tailpipe controls. However, after this initial decline, PM_2.5_ DTT stabilized and gradually increased from ~0.35 in 2012 to ~0.97 nmol/min/m^3^ by 2024, reflecting the growing influence of non-tailpipe emissions such as brake/tire wear. Metals, such as iron (Fe, ~150 ng/m^3^) and zinc (Zn, ~10 ng/m^3^), remained relatively stable as organic and elemental carbon (OC and EC) declined, resulting in non-tailpipe contributions dominating PM_2.5_ toxicity. Although PM_2.5_ mass concentrations were effectively reduced, the growing contribution of non-tailpipe emissions (e.g., brake/tire wear and secondary organic aerosols) underscores the limitations of mass-based standards and tailpipe-focused strategies. Our findings emphasize the need to broaden regulatory strategies, targeting emerging sources that shape PM_2.5_ composition and toxicity and ensuring more improvements in public health outcomes.

## 1. Introduction

Ambient particulate matter (PM) is a critical environmental health concern globally, associated with many adverse health effects, including respiratory and cardiovascular diseases, lung cancer, and increased mortality rates [1,2]. PM is categorized based on aerodynamic diameter into coarse (PM_2.5-10_, 2.5 µm ≤ d_p_ ≤ 10 µm), fine (PM_2.5_, d_p_ ≤ 2.5 µm), and ultrafine particles (UFPs, d_p_ < 0.1 µm) [3,4]. PM_2.5_ poses significant health risks due to its ability to penetrate deep into the lungs, depositing in the alveolar sacs and triggering inflammation and systemic oxidative stress [1,5,6]. Deposited particles translocate into the bloodstream and reach other organs, potentially causing cardiovascular and neurological disorders [7,8,9]. Studies have demonstrated that residents in urban environments where vehicular emissions are the primary source of PM, such as Los Angeles, experience higher incidences of asthma, lung cancer, and cardiovascular diseases compared to those in less polluted areas [10,11,12,13,14]. In the early 2000s, Los Angeles was often subject to frequent air quality alerts, raising concerns among health professionals about the escalating rates of respiratory illnesses, especially among vulnerable populations like children and the elderly [15,16,17]. This public health crisis incited efforts to implement stringent regulatory actions aimed at reducing PM emissions from vehicular and industrial sources [18,19,20].

Over the past decades, a comprehensive set of federal and state-level air quality measures have substantially improved air quality in Los Angeles and across California [20,21,22]. At the federal level, the U.S. Environmental Protection Agency (U.S. EPA) introduced the National Ambient Air Quality Standard (NAAQS) for PM_2.5_ in 1987, aiming to reduce ambient concentrations of PM_2.5_ [23,24]. At the state level, the California Air Resources Board (CARB) introduced stringent vehicular emissions programs with the Low Emission Vehicle I (LEV I) initiative in 1990, later followed by LEV II, LEV III, and the Zero-Emission Vehicle (ZEV) program, each tightening emissions limits on non-methane organic gases (NMOGs), carbon monoxide (CO), nitrogen oxides (NO_x_), and PM [25,26,27]. Additional actions included the Diesel Risk Reduction Plan (2000), financial incentives for replacing high-polluting vehicles (1998–2012), such as the Cleaner Port Truck (CPT) program (2007), Drayage Truck Regulation (2008), and At-Berth Regulation (2010), targeting diesel PM and NO_x_ emissions from freight and maritime activities [28,29,30,31]. In 2013, the Low-Carbon Fuel Standard (LCFS) further encouraged the adoption of cleaner fuels, cutting both particulate and greenhouse gas emissions [32,33]. Hasheminassab et al. (2014) [34] conducted a comprehensive analysis of ambient PM_2.5_ in the Los Angeles Basin from 2002 to 2012, finding a notable reduction in PM_2.5_ concentrations, largely attributed to decreased vehicular emissions resulting from the CARB’s regulations. Similarly, Lurmann et al. (2015) [20] highlighted a 21% reduction in PM_2.5_ and significant decreases in NO_x_ and volatile organic compounds (VOCs) over two decades. These improvements underscore the effectiveness of regulatory measures in mitigating air pollution. However, despite these successes, recent studies indicate that the particle number concentration (PNC) of UFPs has not decreased proportionally and may have plateaued from 2016 onwards [22], suggesting that existing regulations may not sufficiently address UFP emissions, potentially due to the complex nature of UFP sources and formation mechanisms, such as secondary formation and non-tailpipe emissions [35,36]. Moreover, PM comprises a diverse mix of chemical components, including organic compounds, metals, and biological materials, with varying levels of toxicity [37,38,39,40]. Transition metals and polycyclic aromatic hydrocarbons from combustion processes have been associated with higher oxidative potential, posing greater health risks even as overall PM mass declines [41]. Consequently, PM mass concentration alone may not adequately reflect the health impacts, emphasizing the need to consider PM composition and toxicity [42,43,44].

One of the primary mechanisms underlying the adverse health effects of PM exposure is the induction of oxidative stress resulting from interactions with biological systems [45,46]. Oxidative stress occurs when the generation of reactive oxygen species (ROS) exceeds the body’s antioxidant defenses, leading to cellular damage, inflammation, and exacerbation of respiratory and cardiovascular diseases [47,48]. To quantify the oxidative potential of PM, several assays have been developed, with the dithiothreitol (DTT) assay being one of the most widely used [49,50,51,52]. The DTT assay measures the capacity of PM to catalyze electron transfer from DTT to oxygen, simulating redox reactions in biological systems. The rate of DTT consumption correlates with the particle’s ability to generate ROS, serving as an indicator of oxidative potential [53,54]. Studies utilizing the DTT assay have provided valuable insights into the relationship between PM composition and toxicity. Verma et al. (2015) [54] demonstrated that organic aerosols and transition metals significantly contribute to the oxidative potential of PM_2.5_. Despite reductions in PM mass concentrations in Los Angeles, the oxidative potential of PM may not have decreased proportionally, possibly due to shifts in PM composition towards more toxic constituents [21].

The disconnect between trends of PM mass concentration and toxicity underscores an urgent need to reevaluate air quality metrics and regulatory strategies [4,42,43,55]. Relying solely on mass-based standards may not fully consider the increased health risks posed by the oxidative potential of PM. This study aims to investigate the long-term temporal trends in PM toxicity, focusing on the oxidative potential of PM_2.5_ as measured by the DTT assay in Los Angeles, potentially resulting from PM emission regulations. By examining data spanning the last two decades, we sought to understand the influence of regulatory measures on oxidative potential, considering that none of the regulations specifically targets PM toxicity. This analysis fills a critical knowledge gap and provides insights for policymakers, enabling them to adapt and enhance current and future strategies aimed at improving air quality and public health. By focusing on both PM mass and toxicity, we can develop a more comprehensive understanding of the health impacts of air pollution and devise more effective regulatory interventions.

## 2. Methods

### 2.1. Data Collection

Data for PM_2.5_ oxidative potential were collected through a comprehensive review of studies conducted over the past two decades [48,50,56,57,58,59,60,61,62,63,64,65,66,67]. The initially reported DTT data were presented in different units by various studies (i.e., some as activity per mass of PM, others per volume of air) and were converted between these units using the respective PM mass and sampling duration measurements, ensuring that all DTT data could be directly compared on a consistent basis. Moreover, DTT activity per volume was presented in the main text as it more directly reflects inhalation exposure scenarios, providing a more relevant metric of aerosol oxidative potential for environmental and public health assessments compared to mass-normalized values [68]. Table S1 outlines the sampling campaigns and datasets. Most of these studies were carried out using samplers with consistent protocols for measuring PM oxidative potential [48,50,56,57,58,59,60,61,62,63,64,65,66,67,69,70]. Detailed information on measurement methods, equipment calibration, instrument specifications, and uncertainties can be found in the original publications cited in Table S1. Most of the DTT data were sourced from the particle instrumentation unit (PIU) of the University of Southern California (USC), which is located in an urban area near downtown Los Angeles, 150 m downwind of a major freeway (I-110). Additionally, the trends of different chemical species such as metals (i.e., barium (Ba), copper (Cu), manganese (Mn), zinc (Zn), chromium (Cr), nickel (Ni), cadmium (Cd), and iron (Fe)) as well as inorganic ions (i.e., sulfate (SO_4_^2−^) and nitrate (NO_3_^−^) were obtained from the North Main Street monitoring station, operated by the U.S. EPA as part of the Air Quality System (AQS) and Chemical Speciation Network (CSN) [71]. These trends correspond to the sampling periods of the reviewed DTT studies. It should be noted that these trends were only available from 2005 to 2024, as the CSN did not provide data prior to 2005, and no metals data were available in 2005.

### 2.2. Additional Sampling, Instrumentation, and Analysis in 2024

The sampling campaigns for PM_2.5_ were conducted at the PIU of the USC during January and February 2024. For PM_2.5_ collection, pre-baked quartz filters (SKC Inc., Eighty-Four, PA, USA) were used in Sioutas Personal Cascade Impactor Samplers (PCISs, SKC Inc., Eighty-Four, PA, USA), operating with a flow rate set at 9 L per minute [72], to ensure accurate mass measurements. This additional sampling during January–February 2024 was conducted to evaluate contemporary conditions under the latest regulatory environment, using the same DTT measurement protocols as earlier datasets, enabling a comparison with historical trends and highlighting any emerging shifts in PM_2.5_ toxicity. The interpretation of our findings should be contextualized by the temporal limitations of the two-month sampling period, ensuring that conclusions drawn account for potential seasonal variability not captured in this snapshot.

DTT activity serves as a proxy for the oxidative capacity of ambient particles, which may initiate or exacerbate inflammatory processes in humans, and multiple studies have linked higher oxidative potential to elevated cardiopulmonary risks [73,74]. The oxidative potential of collected PM_2.5_ samples was assessed using the DTT assay following established protocols [50,75]. Briefly, PM extracts were incubated with a DTT solution, and the rate of DTT consumption was measured spectrophotometrically, which serves as an indicator of the particle’s capacity to generate ROS. All assays were performed in triplicate and under controlled laboratory conditions to ensure consistency and reliability of the results. While consistent protocols were used to reduce uncertainties, the DTT assay has limitations that may not fully capture all pathways of particle-induced oxidative stress due to factors like incomplete extraction, measurement interferences, and sensitivity to specific species [76]. It should be noted that the DTT data provided for PM_2.5_ DTT in 2008 were obtained using the same methodology as described by Verma et al. (2009) [65]. Pearson correlation analyses (*p* < 0.05) were performed to evaluate associations between DTT activity and chemical species concentrations such as OC/EC and metals. All statistical operations were conducted in Python (version 3.12.0) using the numpy (version 1.26.0) and pandas (version 2.1.1) libraries for data handling and analysis.

## 3. Results and Discussions

### 3.1. Long-Term Trends in PM_2.5_ Composition

**Figure 1:**
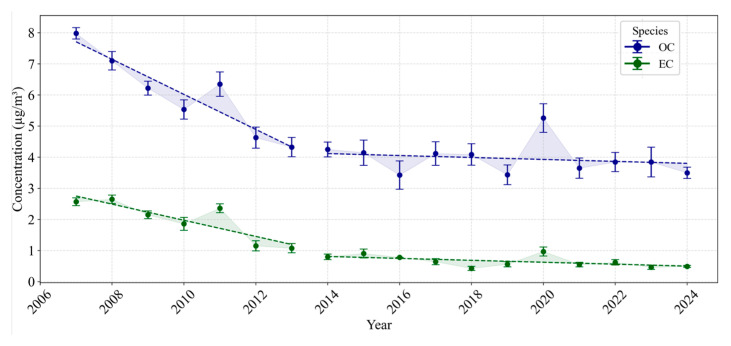
Yearly averaged long-term PM_2.5_ concentrations of OC and EC in Los Angeles (2007–2024).

**Figure 2:**
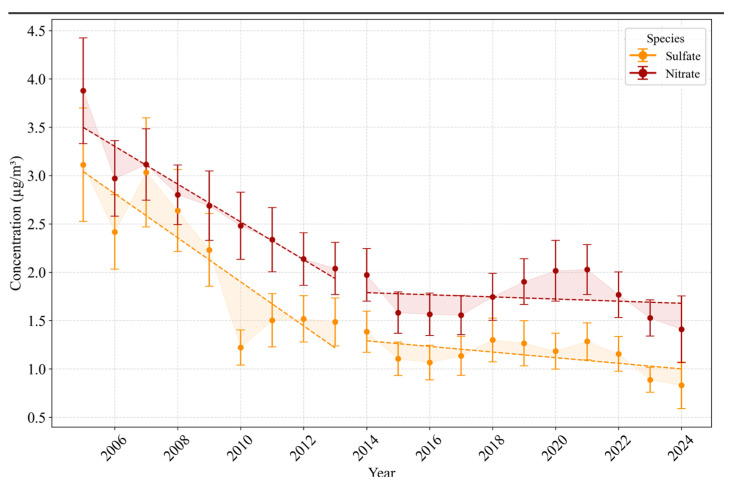
Yearly averaged long-term PM_2.5_ concentrations of sulfate and nitrate in Los Angeles (2005–2024).

**Figure 3:**
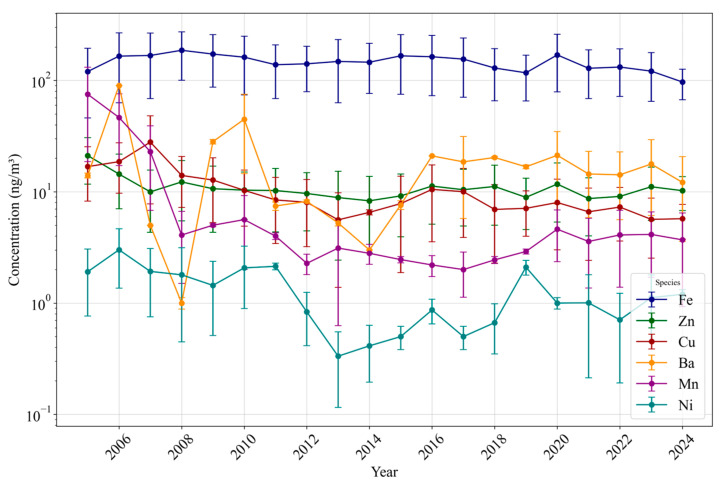
Yearly averaged long-term concentrations of metals in Los Angeles (2007–2024).

This section presents the yearly averaged concentrations of various PM_2.5_ species based on data retrieved from the AQS, as shown in Figure 1, Figure 2 and Figure 3, which illustrate long-term trends in PM_2.5_ components in Los Angeles. Additionally, discrete sampling data were also retrieved from the AQS but were limited to the specific dates provided by prior studies for DTT measurements. These discrete sampling data are presented in the Supplementary Information (Figures S1–S3), providing further context on the evolving PM_2.5_ composition and insights into the specific dates when DTT activity was measured.

#### 3.1.1. Carbonaceous Species: Organic Carbon and Elemental Carbon

Figure 1 presents the yearly averaged concentrations of OC and EC in Los Angeles from 2007 to 2024, revealing two distinct subperiods for both species. During the first subperiod (2007–2016), OC overall decreased from 7.98 µg/m^3^ in 2007 to 3.43 µg/m^3^ by 2016. Despite a brief increase to 6.35 µg/m^3^ in 2011, these marked reductions align with regulatory actions focused on reducing tailpipe emissions [26,30]. This trend aligns with the results of Badami et al. (2024) [22], noting that pollutant reductions were more pronounced during earlier years (2007–2015) due to impactful regulatory efforts targeting primary tailpipe emissions. In contrast, the second subperiod (2017–2024) is characterized by a plateau in OC, varying between approximately 3.43 µg/m^3^ in 2019 and 5.26 µg/m^3^ in 2020 before settling around 3.50 µg/m^3^ in 2024. This stabilization, coupled with intermittent spikes (e.g., the notable increase in 2020 possibly linked to wildfires, including the Bobcat wildfire [77]), suggests that while tailpipe emissions have been effectively controlled, ongoing contributions from non-tailpipe and secondary aerosol sources continue to influence ambient OC levels [78,79]. This trend is consistent with the findings of Badami et al. (2024) [22], who demonstrated a similar plateau in primary pollutant levels since 2016, underscoring the diminished impact of current regulatory measures and the need for stricter standards. Discrete sampling data corresponding to DTT activity, illustrated in Figure S1, generally align with the overall decreasing trends but also reveal shorter-term variations possibly driven by meteorological factors and transient emission episodes, such as wildfires.

EC exhibits a similar two-phase trend over the same period, with concentrations declining significantly from 2.57 µg/m^3^ in 2007 to 0.78 µg/m^3^ in 2016 during the first phase. This marked reduction highlights the success of diesel-specific regulations and fleet modernization in curbing combustion-derived PM_2.5_ emissions [30]. However, beginning in 2017, EC levels stabilized below 1 µg/m^3^ without a consistent downward trend, fluctuating between 0.43 µg/m^3^ in 2018 and 0.97 µg/m^3^ in 2020 before settling in the range of 0.46–0.62 µg/m^3^ by 2024. These findings suggest that residual combustion sources continue to contribute to EC levels, highlighting the need for additional regulatory measures to address these emissions [26]. This trend aligns with observations from Badami et al. (2024) [22], which emphasize the initial effectiveness of regulations followed by a plateau in pollutant levels, underscoring the limitations of existing controls. The concurrent EC measurements corresponding to DTT activity (Figure S1) further support these trends, revealing episodic surges that highlight the interplay between effective tailpipe regulations and transient emission sources. Collectively, the two-phase evolution observed for both OC and EC indicates a sustained decline in primary tailpipe emissions during the earlier years, followed by stabilization in recent years.

#### 3.1.2. Inorganic Ions: Nitrate and Sulfate

Figure 2 illustrates yearly averaged sulfate and nitrate concentrations in Los Angeles from 2005 to 2024, revealing two subperiods consistent with the broader regulatory timeline. During the first subperiod (2005–2016), sulfate decreased from approximately 3.11 µg/m^3^ in 2005 to 1.06 µg/m^3^ in 2016, while nitrate similarly declined from 3.88 µg/m^3^ in 2005 to 1.55 µg/m^3^ by 2016. This substantial reduction aligns with progressively stricter sulfur and nitrogen oxide controls, which target on-road and off-road sources of NO_x_ and SO_x_ [64,80]. In the second subperiod (2016–2024), both sulfate and nitrate remain below their mid-2000s levels but exhibit minor variability. For instance, sulfate concentrations stabilized around 1.50 µg/m^3^ during 2011–2015, followed by a gradual decline to 0.78 µg/m^3^ by 2024. Nitrate similarly stabilizes to values near 1.5–2.0 µg/m^3^ after 2016, with modest variability across the later years. These plateaus suggest that while tailpipe regulations and fuel improvements have significantly curtailed sulfate and nitrate formation pathways, other factors—including regional atmospheric chemistry, wildfires, and potential contributions from stationary sources—can produce episodic elevations [34,65,81].

Additionally, sulfate and nitrate exhibit broadly consistent downward trends in both the annual AQS dataset (Figure 2) and the discrete sampling intervals used in previous DTT-focused studies (Figure S2). This consistency possibly stems from stronger and more direct controls on sulfur content in fuels and NO_x_ emissions from on-road and off-road engines, resulting in relatively stable, long-term reductions in these inorganic ions [82]. In contrast, OC and EC levels display more pronounced fluctuations between the annual and discrete datasets. A significant decrease (*p* < 0.05) in OC, EC, nitrate, and sulfate concentrations was observed during the first subperiod (2007–2014), followed by a plateau from 2014 onwards. This trend mirrors total PM_2.5_ changes in Los Angeles, as corroborated by the work of Badami et al. (2024), affirming that early regulatory efforts were highly effective, with subsequent gains being more modest [22]. The difference arises because EC and especially OC are influenced by a broader range of sources—beyond tailpipe emissions—including mechanical abrasion (e.g., brake and tire wear), secondary organic aerosol formation VOCs, and re-suspended dust [21,83,84]. These non-tailpipe processes are not as directly constrained by existing regulations, leading to less uniform and at times divergent trends when comparing annual means to snapshots aligned with DTT sampling. As a result, even though EC and OC exhibit predominant declines from early regulatory efforts, their more diverse and evolving source profiles yield significant year-to-year variability not seen in the comparatively steadier trajectories of sulfate and nitrate. Moreover, the maritime sector also emits precursors—often referred to as ‘delayed primary organics’—that transform into secondary organic aerosols, boosting oxidative potential even as sulfur-related emissions decline under stricter fuel regulations [85]. Previous studies have demonstrated that port activities, including ships, locomotives, and heavy-duty vehicles, can substantially affect coastal neighborhoods, contributing to elevated OC levels and reinforcing the role of non-tailpipe sources in driving PM_2.5_ toxicity [85,86].

#### 3.1.3. Trace Elements and Metals

Figure 3 illustrates the yearly trends of key trace elements and metal species in Los Angeles between 2005 and 2024, indicating a comparatively stable pattern over this interval. While OC and EC exhibit clear subperiods of decline, the concentrations of metals such as Ba, Cr, Cu, Fe, Mn, Ni, Ti, and Zn remain relatively constant or fluctuate, reflecting non-tailpipe and largely unregulated sources (e.g., brake/tire wear, resuspended road dust, and industrial emissions) [63,87,88]. In particular, Fe shows the highest concentrations, ranging from approximately 100 to 170 ng/m^3^, with an average of almost 150 ng/m^3^ during this period, and occasional spikes, such as ~210 ng/m^3^ in 2020. Other metals, including Ba, Zn, and Cu, exhibit concentrations around 10–20 ng/m^3^, consistent with previous findings in the literature [61,87].

These metals do not exhibit the sustained downward trends observed for combustion-related species, indicating that stricter tailpipe regulations have reduced OC and EC emissions, and metals might have emerged as more important contributors to PM_2.5_ toxicity [64]. Concurrent sampling intervals corresponding to reported DTT activities (Figure S3) support this observation, showing that metal concentrations remain substantial even when OC and EC levels are comparatively low. The stable concentrations of metals, combined with the declining trends of tailpipe emission tracers (e.g., OC and EC), suggest an increasing relative contribution of non-tailpipe metal sources to PM_2.5_ oxidative potential as their proportion in PM_2.5_ mass concentrations continues to grow, which is explored in greater detail in the following sections.

#### 3.1.4. Long-Term Trends of the Ratio of Trace Elements and Metals to Elemental Carbon

**Figure 4:**
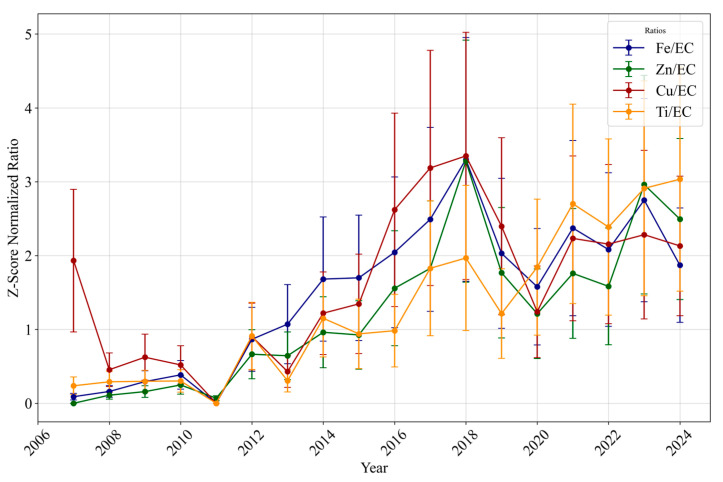
Normalized averaged long-term normalized ratio of specific metals to EC mass in Los Angeles (2007–2024). The error bars represent the standard deviation of the yearly normalized ratios.

To further illustrate the shift from tailpipe to non-tailpipe sources, Figure 4 presents yearly concentration ratios of Fe/EC, Zn/EC, Cu/EC, and Ti/EC from 2007 to 2024, derived from a broad set of annual sampling days rather than only those aligned with DTT measurements. To account for the different scales of mean metals’ concentrations, particularly Fe, compared to others, the ratios were normalized using Shift Z-Score Normalization. This method standardizes the data by subtracting the mean and dividing it by the standard deviation for each metal, ensuring comparability across species. Additionally, a constant equal to the minimum Z-score was added to all normalized values to eliminate negative values.

The normalized Fe/EC ratio, for example, shows a steady increase from approximately 0.47 in 2007 to 3.65 in 2018, maintaining values around 2.0 in the early 2020s. Similar upward trends are observed for Zn/EC and Cu/EC. The earlier regulatory years exhibit significantly lower metal-to-EC ratios, reflecting the dominant influence of tailpipe emissions during that time. The higher Cu/EC ratio for 2007 can be attributed to comparatively elevated Cu concentrations during that year, as shown in Figure 3, and to the ratio’s reliance on Cu data within the Shift Z-Score Normalization process. These rising metal/EC ratios further corroborate the arguments in the previous sections that as regulations effectively reduce tailpipe-related EC emissions in the earlier years, non-tailpipe sources, such as tire and brake wear and re-suspended road dust, contribute an increasing fraction to the PM_2.5_ composition [26,87]. This shift might suggest an increased role of non-tailpipe sources in influencing the oxidative potential of ambient aerosols, as their proportional contribution to PM_2.5_ mass has grown relative to declining tailpipe emissions, which is in agreement with the results reported in the literature [21].

### 3.2. Trends in DTT Activity and Correlations with Species

#### 3.2.1. Long-Term Trends of Oxidative Potential of PM_2.5_

**Figure 5:**
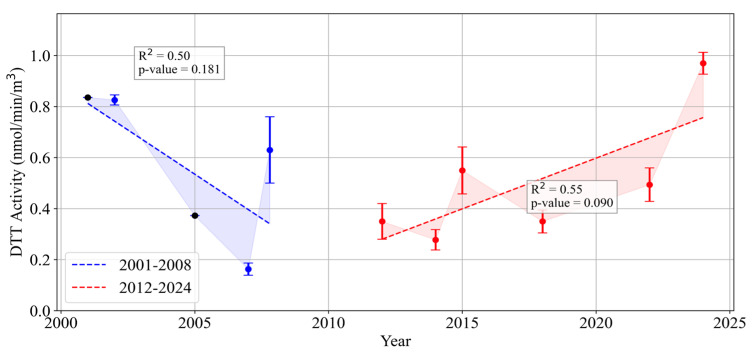
Long-term trends of volumetric DTT activity of PM_2.5_ in Los Angeles (2001–2024).

Figure 5 presents the long-term oxidative potential of PM_2.5_, measured by the DTT activity per volume, from 2001 to 2024, revealing two distinct subperiods. The first subperiod (2001–2008) is characterized by a pronounced decline in DTT activity, which decreased from approximately 0.836 nmol/min/m^3^ in 2001 to 0.163 nmol/min/m^3^ by 2007. This substantial reduction corresponds to the early phase of to the early phase of tailpipe emissions regulatory actions (initiated in 2000) and the implementation of stricter standards under the U.S. EPA [28,29]. These regulatory actions significantly reduced combustion-derived particulate matter, thereby diminishing the oxidative burden associated with tailpipe emissions.

The second subperiod (2012–2024) reveals a gradual rise in DTT activity, increasing from approximately 0.35 nmol/min/m^3^ in 2012 to nearly 0.97 nmol/min/m^3^ by 2024. This trend indicates that while overall PM mass and primary tailpipe tracers such as EC and OC have declined due to ongoing regulatory efforts, alternative sources—particularly non-tailpipe emission tracers (e.g., Fe, Cu, Zn from brake and tire wear) and secondary organic aerosols—have become increasingly significant in influencing PM_2.5_ oxidative potential [61]. These findings align with the concurrent shift in PM composition discussed in Section 3.1.3 and Section 3.1.4, where metals and friction-derived emissions emerged as major contributors to ambient toxicity. As older diesel fleets were retrofitted or replaced and advanced emissions controls were implemented, the relative contribution of non-tailpipe sources grew, reflecting a broader transformation in the composition of urban PM in Los Angeles [80,89]. Consequently, the PM_2.5_ DTT activity per volume highlights an evolving toxicity profile increasingly driven by mechanisms and species not directly targeted by traditional tailpipe-focused regulations.

Figure S4 further complements this analysis by presenting the long-term trend of DTT activity per PM_2.5_ mass in Los Angeles. Similar to the per-volume metric, DTT per mass reveals two subperiods, with an initial decline during the early regulatory years, consistent with the success of early tailpipe-focused measures. However, following this initial reduction, DTT per mass exhibits a much slower rate of decrease, approaching a near-stable or very slow decline in later years. This subtle downward trend suggests a gradual rather than pronounced reduction in intrinsic aerosol toxicity over time.

The distinction between per-volume and per-mass trends highlights a shift in PM_2.5_ composition. The initial reduction in DTT activity per volume reflects regulatory success in reducing the overall ambient PM concentration. However, the slower decline in DTT activity per mass suggests that the composition of the remaining PM has shifted toward a higher relative contribution of more toxic components, such as non-tailpipe metals, secondary organic aerosols, and frictional emissions. This compositional shift underscores the growing proportional role of unregulated sources as primary components like sulfates, nitrates, and EC were reduced. These trends suggest that while overall PM_2.5_ mass concentrations declined due to tailpipe emission controls, the oxidative potency of the remaining PM_2.5_ mass remains significant, driven by emerging non-tailpipe and secondary sources, emphasizing the importance of expanding regulatory efforts to address these sources to further mitigate PM_2.5_ toxicity.

#### 3.2.2. Correlations of Species with PM_2.5_ DTT Activity

**Figure 6:**
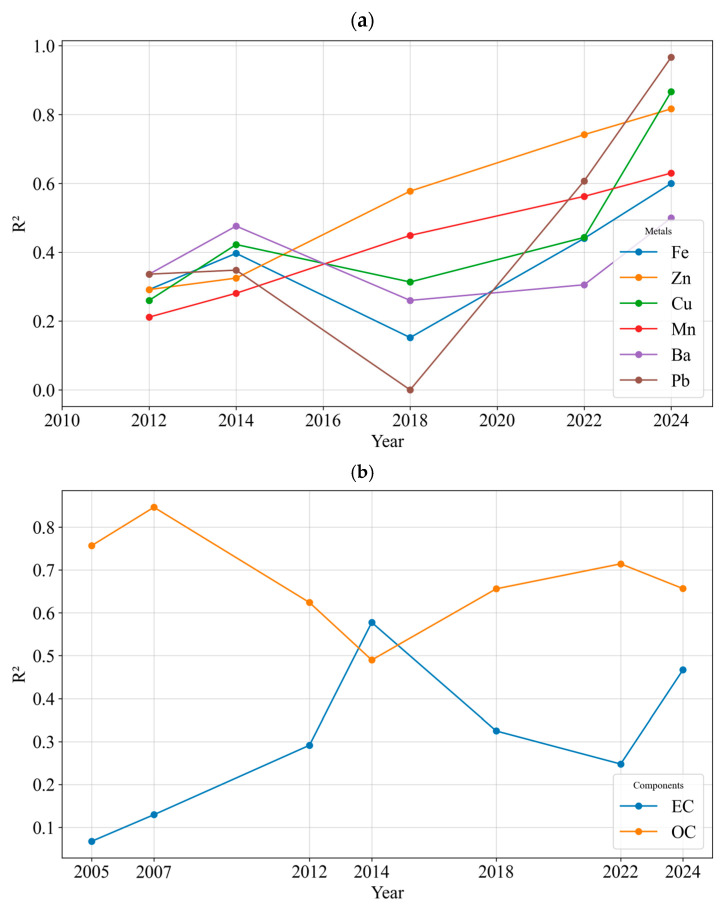
Long-term trends of correlation of (**a**) metals and (**b**) OC and EC with DTT activity of PM_2.5_ in Los Angeles.

Figure 6a,b presents long-term trends in the correlations between DTT activity and key PM_2.5_ constituents, including EC, OC, Fe, Zn, Cu, Mn, and Pb, based on data collected in Los Angeles from 2012 to 2024. We examined the correlation between individual chemical species and volumetric DTT activity to highlight the key contributors to PM_2.5_-induced oxidative stress [90]. Only statistically significant correlations (*p* < 0.05) are shown in the figure. Over time, the correlations between metals (e.g., Fe, Zn, Cu, Mn, and Pb) and DTT activity tend to strengthen, consistent with earlier studies that identified transition metals as major drivers of PM oxidative potential [53,73,91]. By the 2015–2018 period, correlation coefficients for these metals were notably higher, reflecting the increasing contribution of non-tailpipe metal emissions to the oxidative potential of fine PM as tailpipe-related sources diminished under the influence of regulatory measures [87,92]. Although the 2018 dataset [57] included averages from multiple global cities—potentially introducing some variability not solely attributable to local factors—the overall pattern still aligns with the observed decline in combustion-related influences and the growing significance of non-tailpipe sources, such as brake and tire wear, as key contributors to PM_2.5_ toxicity.

For OC and EC, contrasting trends are observed in their correlations with DTT activity. OC exhibits a slight decreasing trend in correlation, which may indicate the reduced role of tailpipe emissions in driving the oxidative potential of fine PM over time [21]. Moreover, OC consistently displays stronger correlations with DTT activity compared to EC, with the exception of one year, in agreement with the previous studies [92]. This suggests that OC has a greater intrinsic role in contributing to oxidative potential. In contrast, EC shows a modest increase in its correlation with DTT activity, a trend possibly driven by its concurrent emissions with non-tailpipe tracers, such as metals, which are strongly correlated with the oxidative potential of fine PM, rather than its own inherent toxicity. The rising correlation of EC with DTT activity may therefore reflect the indirect influence of non-tailpipe sources on oxidative potential. These trends underscore the growing importance of emissions from non-tailpipe emissions and resuspension processes in the modern PM_2.5_ chemical composition in megacities such as Los Angeles [21]. Despite the slight decrease trend of OC levels and its correlations with the oxidative potential of fine PM, it still remains high, highlighting the importance of other sources of OC, such as photochemistry, biomass combustion, and non-tailpipe emissions, in addition to primary emissions that are also part of OC [21].

Moreover, the modern vehicle fleet exhibits shifting patterns of UFP emissions related to emerging technologies and after-treatment systems. While diesel emissions have dropped due to widespread adoption of diesel particulate filters (DPFs), gasoline direct injection (GDI) engines emit greater numbers of UFPs and larger quantities of semi-volatile or intermediate-volatility organic compounds (IVOCs and SVOCs) that foster secondary organic aerosol formation [93]. In diesel vehicles, oxidation catalysts (DOCs) effectively reduce hydrocarbon emissions but also produce sulfuric acid by oxidizing SO₂, which can nucleate with oil-derived organics to form nanoparticles [94]. Although DPFs remove most primary soot, they also reduce certain precursors of secondary organic aerosols [95]. Consequently, modern diesel engines can have reduced PM mass yet still feature nanoparticle formation pathways involving delayed organics—much of which originate from lubricating oil [96]. These organic-rich nanoparticles and associated metal additives likely account for a portion of the residual oxidative potential observed in ambient PM_2.5_, highlighting the need to address both diesel and gasoline fleets as well as non-tailpipe sources in toxicity-oriented regulatory frameworks.

## 4. Summary and Conclusions

This study examined long-term trends in both the composition and oxidative potential of PM_2.5_ in Los Angeles over the past two decades, with a particular focus on the DTT assay as an indicator of particle-induced oxidative stress. Our results demonstrate that although mass concentrations of key combustion markers (e.g., OC and EC) have markedly declined—particularly before 2016—there has been a subsequent shift in PM_2.5_ composition toward non-tailpipe emissions tracers. These species, which remained largely unregulated, accounted for a growing fraction of PM_2.5_ toxicity. This transition was reflected in the partial resurgence or plateau of DTT activity per mass between 2012 and 2024 and the increase in the per volume DTT activity of fine PM, as well as the increasing correlation trends. Metals including Fe, Cu, and Zn remained relatively stable or even proportionally increased within the PM_2.5_ composition, underscoring their elevated oxidative potential in a context where traditional tailpipe emissions have already been substantially mitigated. The growing contribution of non-tailpipe emissions and secondary aerosol formation suggests that residual toxicity is increasingly driven by unregulated sources. Furthermore, while DPFs have successfully lowered PM mass, GDI technology and certain diesel after-treatment processes (e.g., oxidation catalysts) promote nanoparticle formation by enabling the nucleation of sulfuric acid and oil-derived organics. These organic-rich UFPs and associated trace metals, originating partly from lubricating oil, sustain or elevate residual oxidative potential in modern vehicle exhaust, reinforcing the need to regulate both diesel and gasoline sources through toxicity-focused strategies. These findings suggest prioritizing unregulated sources—particularly non-tailpipe metals and secondary organics—will be crucial for further improving air quality and protecting public health. Policymakers could consider integrating toxicity metrics like oxidative potential (e.g., DTT activity) alongside mass concentration limits in future regulatory frameworks to target both total PM mass and its most hazardous chemical components. Our findings suggest that focusing solely on mass-based regulatory standards could overlook toxicologically significant shifts in PM composition, especially the rising influence of metal-rich, non-tailpipe sources. To address these emerging toxicity concerns, policymakers can consider placing tighter limits on metallic additives in brake components, particularly copper and zinc, which continue to appear prominently in PM_2.5_. More stringent guidelines or incentive-based programs for low-wear brake and tire materials would help reduce the fraction of non-tailpipe emissions driving oxidative stress.

## Data Availability

Dataset available on request from the authors.

## Supplementary Materials

The following supporting information can be downloaded at: https://www.mdpi.com/article/10.3390/toxics13020140/s1, Figure S1: OC and EC concentrations in Los Angeles (2007–2024) on dates corresponding to DTT activity reported in prior studies; Figure S2: Sulfate and Nitrate concentrations in Los Angeles (2007–2024) on dates corresponding to DTT activity reported in prior studies; Figure S3: Metals’ concentrations in Los Angeles (2007–2024) on dates corresponding to DTT activity reported in prior studies; Figure S4: Long-term trends of DTT activity per mass of PM_2.5_ in Los Angeles (2001–2024); Table S1: List of studies reporting the DTT activity in Los Angeles from 2001 to 2024; [48,50,56,57,58,59,61,63,64,65].

## References

[1] Brook R.D., Rajagopalan S., Pope C.A., Brook J.R., Bhatnagar A., Diez-Roux A.V., Holguin F., Hong Y., Luepker R.V., Mittleman M.A. (2010). Particulate matter air pollution and cardiovascular disease: An update to the scientific statement from the american heart association. Circulation.

[2] Pope C.A., Dockery D.W. (2006). Health effects of fine particulate air pollution: Lines that connect. J. Air Waste Manag. Assoc..

[3] Becker S., Soukup J.M. (2003). Coarse (PM2.5-10), fine (PM2.5), and ultrafine air pollution particles induce/increase immune costimulatory receptors on human blood-derived monocytes but not on alveolar macrophages. J. Toxicol. Environ. Health—Part A.

[4] WHO WHO Global Air Quality Guidelines: Particulate Matter (PM2.5 and PM10), Ozone, Nitrogen Dioxide, Sulfur Dioxide and Carbon Monoxide: Executive Summary. https://www.who.int/publications/i/item/9789240034433.

[5] Willers S.M., Eriksson C., Gidhagen L., Nilsson M.E., Pershagen G., Bellander T. (2013). Fine and coarse particulate air pollution in relation to respiratory health in Sweden. Eur. Respir. J..

[6] Zhang Y., Liu L., Zhang L., Yu C., Wang X., Shi Z., Hu J., Zhang Y. (2022). Assessing short-term impacts of PM2.5 constituents on cardiorespiratory hospitalizations: Multi-city evidence from China. Int. J. Hyg. Environ. Health.

[7] Kreyling W.G., Semmler-Behnke M., Möller W. (2006). Ultrafine particle-lung interactions: Does size matter?. J. Aerosol. Med..

[8] Oberdörster G. (2000). Pulmonary effects of inhaled ultrafine particles. Int. Arch. Occup. Environ. Health.

[9] Oberdörster G., Oberdörster E., Oberdörster J. (2005). Nanotoxicology: An emerging discipline evolving from studies of ultrafine particles. Environ. Health Perspect..

[10] Burnett R., Chen H., Szyszkowicz M., Fann N., Hubbell B., Pope C.A., Apte J.S., Brauer M., Cohen A., Weichenthal S. (2018). Global estimates of mortality associated with long-term exposure to outdoor fine particulate matter. Proc. Natl Acad. Sci. USA.

[11] Crouse D.L., Peters P.A., van Donkelaar A., Goldberg M.S., Villeneuve P.J., Brion O., Khan S., Atari D.O., Jerrett M., Pope C.A. (2012). Risk of nonaccidental and cardiovascular mortality in relation to long-term exposure to low concentrations of fine particulate matter: A canadian national-level cohort study. Environ. Health Perspect..

[12] Jerrett M., Burnett R.T., Beckerman B.S., Turner M.C., Krewski D., Thurston G., Martin R.V., Donkelaar A.V., Hughes E., Shi Y. (2013). Spatial analysis of air pollution and mortality in California. Am. J. Respir. Crit. Care Med..

[13] Künzli N., Jerrett M., Mack W.J., Beckerman B., LaBree L., Gilliland F., Thomas D., Peters J., Hodis H.N. (2005). Ambient air pollution and atherosclerosis in Los Angeles. Environ. Health Perspect..

[14] Pope C.A., Burnett R.T., Thun M.J., Calle E.E., Krewski D., Ito K., Thurston G.D. (2002). Lung cancer, cardiopulmonary mortality, and long-term exposure to fine particulate air pollution. J. Am. Med. Assoc..

[15] Gilliland F.D., Berhane K., Rappaport E.B., Thomas D.C., Avol E., Gauderman W.J., London S.J., Margolis H.G., McConnell R., Islam K.T. (2001). The effects of ambient air pollution on school absenteeism due to respiratory illnesses. Epidemiology.

[16] Jerrett M., Burnett R.T., Ma R., Pope C.A., Krewski D., Newbold K.B., Thurston G., Shi Y., Finkelstein N., Calle E.E. (2005). Spatial analysis of air pollution and mortality in Los Angeles. Epidemiology.

[17] McConnell R., Berhane K., Gilliland F., London S.J., Islam T., Gauderman W.J., Avol E., Margolis H.G., Peters J.M. (2002). Asthma in exercising children exposed to ozone: A cohort study. Lancet.

[18] CARB Almanac of Emissions & Air Quality|California Air Resources Board. https://ww2.arb.ca.gov/our-work/programs/almanac-emissions-air-quality.

[19] Lloyd A.C., Cackette T.A. (2001). Diesel Engines: Environmental Impact and Control. J. Air Waste Manag. Assoc..

[20] Lurmann F., Avol E., Gilliland F. (2015). Emissions reduction policies and recent trends in Southern California’s ambient air quality. J. Air Waste Manag. Assoc..

[21] Altuwayjiri A., Pirhadi M., Taghvaee S., Sioutas C. (2021). Long-Term trends in the contribution of PM2.5sources to organic carbon (OC) in the Los Angeles basin and the effect of PM emission regulations. Faraday Discuss..

[22] Badami M.M., Tohidi R., Sioutas C. (2024). Los Angeles Basin’s air quality transformation: A long-term investigation on the impacts of PM regulations on the trends of ultrafine particles and co-pollutants. J. Aerosol Sci..

[23] Cheung K., Shafer M.M., Schauer J.J., Sioutas C. (2012). Historical trends in the mass and chemical species concentrations of coarse particulate matter in the Los Angeles basin and relation to sources and air quality regulations. J. Air Waste Manag. Assoc..

[24] Suh H.H., Bahadori T., Vallarino J., Spengler J.D. (2000). Criteria air pollutants and toxic air-pollutants. Environ. Health Perspect..

[25] CARB Low-Emission Vehicle Program|California Air Resources Board. https://ww2.arb.ca.gov/our-work/programs/low-emission-vehicle-program/about.

[26] Singh S., Kulshrestha M.J., Rani N., Kumar K., Sharma C., Aswal D.K. (2023). An Overview of Vehicular Emission Standards. Mapan.

[27] Warneke C., Gouw J.A.D., Holloway J.S., Peischl J., Ryerson T.B., Atlas E., Blake D., Trainer M., Parrish D.D. (2012). Multiyear trends in volatile organic compounds in Los Angeles, California: Five decades of decreasing emissions. J. Geophys. Res. Atmos..

[28] CARB AB 32 Climate Change Scoping Plan|California Air Resources Board. https://ww2.arb.ca.gov/our-work/programs/ab-32-climate-change-scoping-plan.

[29] Goodchild A., Mohan K. (2008). The clean trucks program: Evaluation of policy impacts on marine terminal operations. Marit. Econ. Logist..

[30] Haveman J., Thornberg C. (2008). Clean Trucks Program-3. https://www.researchgate.net/publication/265047713_CLEAN_TRUCKS_PROGRAM_AN_ECONOMIC_POLICY_ANALYSIS_Prepared_by.

[31] Lee G., You S.I., Ritchie S.G., Saphores J.D., Jayakrishnan R., Ogunseitan O. (2012). Assessing air quality and health benefits of the Clean Truck Program in the Alameda corridor, CA. Transp. Res. Part A Policy Pract..

[32] Andress D., Nguyen T.D., Das S. (2010). Low-carbon fuel standard-Status and analytic issues. Energy Policy.

[33] CARB Low Carbon Fuel Standard|California Air Resources Board. https://ww2.arb.ca.gov/our-work/programs/low-carbon-fuel-standard.

[34] Hasheminassab S., Daher N., Ostro B.D., Sioutas C. (2014). Long-term source apportionment of ambient fine particulate matter (PM 2.5) in the Los Angeles Basin: A focus on emissions reduction from vehicular sources. Environ. Pollut..

[35] Gani S., Chambliss S.E., Messier K.P., Lunden M.M., Apte J.S. (2021). Spatiotemporal profiles of ultrafine particles differ from other traffic-related air pollutants: Lessons from long-term measurements at fixed sites and mobile monitoring. Environ. Sci. Atmos..

[36] Kumar P., Pirjola L., Ketzel M., Harrison R.M. (2013). Nanoparticle emissions from 11 non-vehicle exhaust sources—A review. Atmos. Environ..

[37] Adamson I.Y.R., Prieditis H., Hedgecock C., Vincent R. (2000). Zinc is the toxic factor in the lung response to an atmospheric particulate sample. Toxicol. Appl. Pharmacol..

[38] Bello D., Hsieh S.F., Schmidt D., Rogers E. (2009). Nanomaterials properties vs. biological oxidative damage: Implications for toxicity screening and exposure assessment. Nanotoxicology.

[39] Gualtieri M., Øvrevik J., Holme J.A., Perrone M.G., Bolzacchini E., Schwarze P.E., Camatini M. (2010). Differences in cytotoxicity versus pro-inflammatory potency of different PM fractions in human epithelial lung cells. Toxicol Vitr..

[40] Niu X., Wang Y., Chuang H.C., Shen Z., Sun J., Cao J., Ho K.F. (2022). Real-time chemical composition of ambient fine aerosols and related cytotoxic effects in human lung epithelial cells in an urban area. Environ. Res..

[41] Shuster-Meiseles T., Shafer M.M., Heo J., Pardo M., Antkiewicz D.S., Schauer J.J., Rudich A., Rudich Y. (2016). ROS-generating/ARE-activating capacity of metals in roadway particulate matter deposited in urban environment. Environ. Res..

[42] Bates J.T., Fang T., Verma V., Zeng L., Weber R.J., Tolbert P.E., Abrams J.Y., Sarnat S.E., Klein M., Mulholland J.A. (2019). Review of Acellular Assays of Ambient Particulate Matter Oxidative Potential: Methods and Relationships with Composition, Sources, and Health Effects. Environ. Sci. Technol..

[43] Kelly F.J., Fussell J.C. (2012). Size, source and chemical composition as determinants of toxicity attributable to ambient particulate matter. Atmos. Environ..

[44] Alramzi Y., Aghaei Y., Badami M.M., Aldekheel M., Tohidi R., Sioutas C. Investigating Urban Emission and Lung-Deposited Surface Area Sources and Their Diurnal Trends in Fine and Ultrafine Pm in Los Angeles 2024. https://papers.ssrn.com/sol3/papers.cfm?abstract_id=4916754.

[45] Ayres J.G., Borm P., Cassee F.R., Castranova V., Donaldson K., Ghio A., Harrison R.M., Hider R., Kelly F., Kooter I.M. (2008). Evaluating the toxicity of airborne particulate matter and nanoparticles by measuring oxidative stress potential—A workshop report and consensus statement. Inhal. Toxicol..

[46] Miller M.R., Shaw C.A., Langrish J.P. (2012). From particles to patients: Oxidative stress and the cardiovascular effects of air pollution. Future Cardiol.

[47] Gurgueira S.A., Lawrence J., Coull B., Murthy G.G.K., González-Flecha B. (2002). Rapid increases in the steady-state concentration of reactive oxygen species in the lungs and heart after particulate air pollution inhalation. Environ. Health Perspect..

[48] Li N., Sioutas C., Cho A., Schmitz D., Misra C., Sempf J., Wang M., Oberley T., Froines J., Nel A. (2003). Ultrafine particulate pollutants induce oxidative stress and mitochondrial damage. Environ. Health Perspect..

[49] Charrier J.G., Anastasio C. (2012). On dithiothreitol (DTT) as a measure of oxidative potential for ambient particles: Evidence for the importance of soluble \newline transition metals. Atmos. Chem. Phys..

[50] Cho A.K., Sioutas C., Miguel A.H., Kumagai Y., Froines J.R. (2005). Redox activity of airborne particulate matter (PM) at different sites in the Los Angeles Basin. Environ. Res..

[51] Fang T., Verma V., Guo H., King L.E., Edgerton E.S., Weber R.J. (2015). A semi-automated system for quantifying the oxidative potential of ambient particles in aqueous extracts using the dithiothreitol (DTT) assay: Results from the Southeastern Center for Air Pollution and Epidemiology (SCAPE). Atmos. Meas. Tech..

[52] Gao D., Fang T., Verma V., Zeng L., Weber R.J. (2017). A method for measuring total aerosol oxidative potential (OP) with the dithiothreitol (DTT) assay and comparisons between an urban and roadside site of water-soluble and total OP. Atmos. Meas. Tech..

[53] Fang T., Verma V., Bates J.T., Abrams J., Klein M., Strickland J.M., Sarnat E.S., Chang H.H., Mulholland A.J., Tolbert E.P. (2016). Oxidative potential of ambient water-soluble PM2.5 in the southeastern United States: Contrasts in sources and health associations between ascorbic acid (AA) and dithiothreitol (DTT) assays. Atmos. Chem. Phys..

[54] Verma V., Fang T., Xu L., Peltier R.E., Russell A.G., Ng N.L., Weber R.J. (2015). Organic aerosols associated with the generation of reactive oxygen species (ROS) by water-soluble PM2.5. Environ. Sci. Technol..

[55] US EPA Integrated Science Assessment (ISA) for Particulate Matter. https://www.epa.gov/isa/integrated-science-assessment-isa-particulate-matter.

[56] Shirmohammadi F., Wang D., Hasheminassab S., Verma V., Schauer J.J., Shafer M.M., Sioutas C. (2017). Oxidative potential of on-road fine particulate matter (PM2.5) measured on major freeways of Los Angeles, CA, and a 10-year comparison with earlier roadside studies. Atmos. Environ..

[57] Badami M.M., Tohidi R., Aldekheel M., Farahani V.J., Verma V., Sioutas C. (2023). Design, optimization, and evaluation of a wet electrostatic precipitator (ESP) for aerosol collection. Atmos. Environ..

[58] Farahani V.J., Altuwayjiri A., Pirhadi M., Verma V., Ruprecht A.A., Diapouli E., Eleftheriadis K., Sioutas C. (2022). The oxidative potential of particulate matter (PM) in different regions around the world and its relation to air pollution sources. Environ. Sci. Atmos..

[59] Hu S., Polidori A., Arhami M., Shafer M.M., Schauer J.J., Cho A., Sioutas C. (2008). Redox activity and chemical speciation of size fractioned PM in the communities of the Los Angeles-Long Beach harbor. Atmos. Chem. Phys..

[60] Li N., Wang M., Bramble L.A., Schmitz D.A., Schauer J.J., Sioutas C., Harkema J.R., Nel A.E. (2009). The adjuvant effect of ambient particulate matter is closely reflected by the particulate oxidant potential. Environ. Health Perspect..

[61] Ntziachristos L., Froines J.R., Cho A.K., Sioutas C. (2007). Relationship between redox activity and chemical speciation of size-fractionated particulate matter. Part. Fibre Toxicol..

[62] Saffari A., Daher N., Shafer M.M., Schauer J.J., Sioutas C. (2014). Seasonal and spatial variation in dithiothreitol (DTT) activity of quasi-ultrafine particles in the Los Angeles Basin and its association with chemical species. J. Environ. Sci. Health—Part A Toxic/Hazard. Subst. Environ. Eng..

[63] Saffari A., Hasheminassab S., Shafer M.M., Schauer J.J., Chatila T.A., Sioutas C. (2016). Nighttime aqueous-phase secondary organic aerosols in Los Angeles and its implication for fine particulate matter composition and oxidative potential. Atmos. Environ..

[64] Shirmohammadi F., Hasheminassab S., Wang D., Schauer J.J., Shafer M.M., Delfino R.J., Sioutas C. (2016). The relative importance of tailpipe and non-tailpipe emissions on the oxidative potential of ambient particles in Los Angeles, CA. Faraday Discuss..

[65] Verma V., Polidori A., Schauer J.J., Shafer M.M., Cassee F.R., Sioutas C. (2009). Physicochemical and toxicological profiles of particulate matter in Los Angeles during the October 2007 Southern California wildfires. Environ. Sci. Technol..

[66] Verma V., Pakbin P., Cheung K.L., Cho A.K., Schauer J.J., Shafer M.M., Kleinman M.T., Sioutas C. (2011). Physicochemical and oxidative characteristics of semi-volatile components of quasi-ultrafine particles in an urban atmosphere. Atmos. Environ..

[67] Zhang X., Staimer N., Gillen D.L., Tjoa T., Schauer J.J., Shafer M.M., Hasheminassab S., Pakbin P., Vaziri N.D., Sioutas C. (2016). Associations of oxidative stress and inflammatory biomarkers with chemically-characterized air pollutant exposures in an elderly cohort. Environ. Res..

[68] Yao K., Wang S., Zheng H., Zhang X., Wang Y., Chi Z., Guo H. (2023). Oxidative potential and source apportionment of size-resolved particles from indoor environments: Dithiothreitol (DTT) consumption and ROS production. Atmos. Environ..

[69] Versatile Aerosol Concentration Enrichment System (VACES) for Simultaneous In Vivo and In Vitro Evaluation of Toxic Effects of Ultrafine, Fine and Coarse Ambient Particles Part I: Development and Laboratory Characterization—ScienceDirect. https://www.sciencedirect.com/science/article/pii/S002185020100057X.

[70] Singh M., Misra C., Sioutas C. (2003). Field evaluation of a personal cascade impactor sampler (PCIS). Atmos. Environ..

[71] EPA EPA: Air Quality System (AQS) API. https://scholar.google.com/scholar_lookup?title=Air%20quality%20System%20.

[72] Misra C., Singh M., Shen S., Sioutas C., Hall P.M. (2002). Development and evaluation of a personal cascade impactor sampler (PCIS). J. Aerosol Sci..

[73] Jiang H., Ahmed C.M.S., Canchola A., Chen J.Y., Lin Y.-H. (2019). Use of Dithiothreitol Assay to Evaluate the Oxidative Potential of Atmospheric Aerosols. Atmosphere.

[74] Abrams J.Y., Weber R.J., Klein M., Samat S.E., Chang H.H., Strickland M.J., Verma V., Fang T., Bates J.T., Mulholland J.A. (2017). Associations between Ambient Fine Particulate Oxidative Potential and Cardiorespiratory Emergency Department Visits. Environ. Health Perspect..

[75] Kumagai Y., Koide S., Taguchi K., Endo A., Nakai Y., Yoshikawa T., Shimojo N. (2002). Oxidation of proximal protein sulfhydryls by phenanthraquinone, a component of diesel exhaust particles. Chem. Res. Toxicol..

[76] Verma V., Fang T., Guo H., King L., Bates J.T., Peltier R.E., Edgerton E., Russell A.G., Weber R.J. (2014). Reactive oxygen species associated with water-soluble PM2.5 in the southeastern United States: Spatiotemporal trends and source apportionment. Atmos. Chem. Phys..

[77] Sannigrahi S., Zhang Q., Pilla F., Basu B., Basu A.S. (2020). Effects of West Coast forest fire emissions on atmospheric environment: A coupled satellite and ground-based assessment 2020. arXiv.

[78] Limbeck A., Kulmala M., Puxbaum H. (2003). Secondary organic aerosol formation in the atmosphere via heterogeneous reaction of gaseous isoprene on acidic particles. Geophys. Res. Lett..

[79] Aghaei Y., Badami M.M., Aldekheel M., Tohidi R., Sioutas C. (2025). Seasonal characterization of primary and secondary sources of fine PM-bound water-soluble organic carbon in central Los Angeles. Atmos. Environ..

[80] Tohidi R., Altuwayjiri A., Sioutas C. (2022). Investigation of organic carbon profiles and sources of coarse PM in Los Angeles. Environ. Pollut..

[81] Soleimanian E., Mousavi A., Taghvaee S., Shafer M.M., Sioutas C. (2020). Impact of secondary and primary particulate matter (PM) sources on the enhanced light absorption by brown carbon (BrC) particles in central Los Angeles. Sci. Total Environ..

[82] Graham L.A., Belisle S.L., Rieger P. (2009). Nitrous oxide emissions from light duty vehicles. Atmos. Environ..

[83] Blanchard C.L., Shaw S.L., Edgerton E.S., Schwab J.J. (2021). Ambient PM2.5 organic and elemental carbon in New York City: Changing source contributions during a decade of large emission reductions. J. Air Waste Manag. Assoc..

[84] Murphy B.N., Sonntag D., Seltzer K.M., Pye H.O.T., Allen C., Murray E., Toro C., Gentner D.R., Huang C., Jathar S. (2023). Reactive organic carbon air emissions from mobile sources in the United States. Atmos. Chem. Phys..

[85] Mousavi A., Sowlat M.H., Hasheminassab S., Polidori A., Shafer M.M., Schauer J.J., Sioutas C. (2019). Impact of emissions from the Ports of Los Angeles and Long Beach on the oxidative potential of ambient PM0.25 measured across the Los Angeles County. Sci. Total Environ..

[86] Donateo A., Gregoris E., Gambaro A., Merico E., Giua R., Nocioni A., Contini D. (2014). Contribution of harbour activities and ship traffic to PM2.5, particle number concentrations and PAHs in a port city of the Mediterranean Sea (Italy). Environ. Sci. Pollut. Res..

[87] Farahani V.J., Soleimanian E., Pirhadi M., Sioutas C. (2021). Long-term trends in concentrations and sources of PM2.5–bound metals and elements in central Los Angeles. Atmos. Environ..

[88] Panko J.M., Hitchcock K.M., Fuller G.W., Green D. (2019). Evaluation of Tire Wear Contribution to PM2.5 in Urban Environments. Atmosphere.

[89] CARB Advanced Clean Trucks|California Air Resources Board. https://ww2.arb.ca.gov/our-work/programs/advanced-clean-trucks.

[90] Fang T., Guo H., Verma V., Peltier R.E., Weber R.J. (2015). PM2.5 water-soluble elements in the southeastern United States: Automated analytical method development, spatiotemporal distributions, source apportionment, and implications for heath studies. Atmos. Chem. Phys..

[91] Fujitani Y., Furuyama A., Tanabe K., Hirano S. (2017). Comparison of Oxidative Abilities of PM2.5 Collected at Traffic and Residential Sites in Japan. Contribution of Transition Metals and Primary and Secondary Aerosols. Aerosol Air Qual. Res..

[92] Campbell S.J., Wolfer K., Utinger B., Westwood J., Zhang Z.-H., Bukowiecki N., Steimer S.S., Vu T.V., Xu J., Straw N. (2021). Atmospheric conditions and composition that influence PM_2.5_ oxidative potential in Beijing, China. Atmos. Chem. Phys..

[93] Bessagnet B., Allemand N., Putaud J.-P., Couvidat F., André J.-M., Simpson D., Pisoni E., Murphy B.N., Thunis P. (2022). Emissions of Carbonaceous Particulate Matter and Ultrafine Particles from Vehicles—A Scientific Review in a Cross-Cutting Context of Air Pollution and Climate Change. Appl. Sci..

[94] Pirjola L., Karl M., Rönkkö T., Arnold F. (2015). Model studies of volatile diesel exhaust particle formation: Are organic vapours involved in nucleation and growth?. Atmos. Chem. Phys..

[95] Karjalainen P., Rönkkö T., Simonen P., Ntziachristos L., Juuti P., Timonen H., Teinilä K., Saarikoski S., Saveljeff H., Lauren M. (2019). Strategies To Diminish the Emissions of Particles and Secondary Aerosol Formation from Diesel Engines. Environ. Sci. Technol..

[96] Fushimi A., Saitoh K., Fujitani Y., Hasegawa S., Takahashi K., Tanabe K., Kobayashi S. (2011). Organic-rich nanoparticles (diameter: 10–30 nm) in diesel exhaust: Fuel and oil contribution based on chemical composition. Atmos. Environ..

