# Supplementary material for: Impact of Emission Standards on Fine Particulate Matter Toxicity: A Long-Term Analysis in Los Angeles
Source: Toxics. 2025 Feb 18;13(2):140. doi: 10.3390/toxics13020140 (PMC11861624; doi:10.3390/toxics13020140)
Supplement: Supplementary file 1 [file toxics-13-00140-s001.zip › Supplementary info.pdf]

Article

Impact of Emission Standards on Fine Particulate Matter Toxicity: A Long-Term Analysis in Los Angeles

Mohammad Mahdi Badami, Yashar Aghaei and Constantinos Sioutas *

Department of Civil and Environmental Engineering, University of Southern California, Los Angeles, CA 90089, USA; mbadami@usc.edu (M.M.B.); yaghaei@usc.edu (Y.A.)
* Correspondence: sioutas@usc.edu

Table S1. List of studies reporting the DTT activity in Los Angeles from 2001 to 2024

Year of Sampling	Sampling Period	Location	Source
2001-02	November 2001-March 2002	PIU, USC	Li et al. (2003)
2002-03	January and March 2002-January 2003	Main campus, USC	Cho et al. (2005)
2005	January	PIU, USC	Ntziachristos et al. (2008)
2007	March to May	PIU, USC	Hu et al. (2008)
2007-08	October to November	PIU, USC	Verma et al. (2009)
2008	June to Augest	PIU, USC	Current study
2012-13	July 2012 to February 2013	PIU, USC	Shirmohammadi et al. (2016)
2014-16	October 2014 to January 2015-November 2015 to January 2016	Main campus, USC	Shirmohammadi et al. (2017)
2014-15	August to October 2014-December 2014 to February 2015	PIU, USC	Saffari et al. (2016)
2018-19	August 2018-December 2018 to January 2019	PIU, USC	Farahani et al. (2022)
2022	December	PIU, USC	Badami et al. (2023)
2024	January to February	PIU, USC	Current study

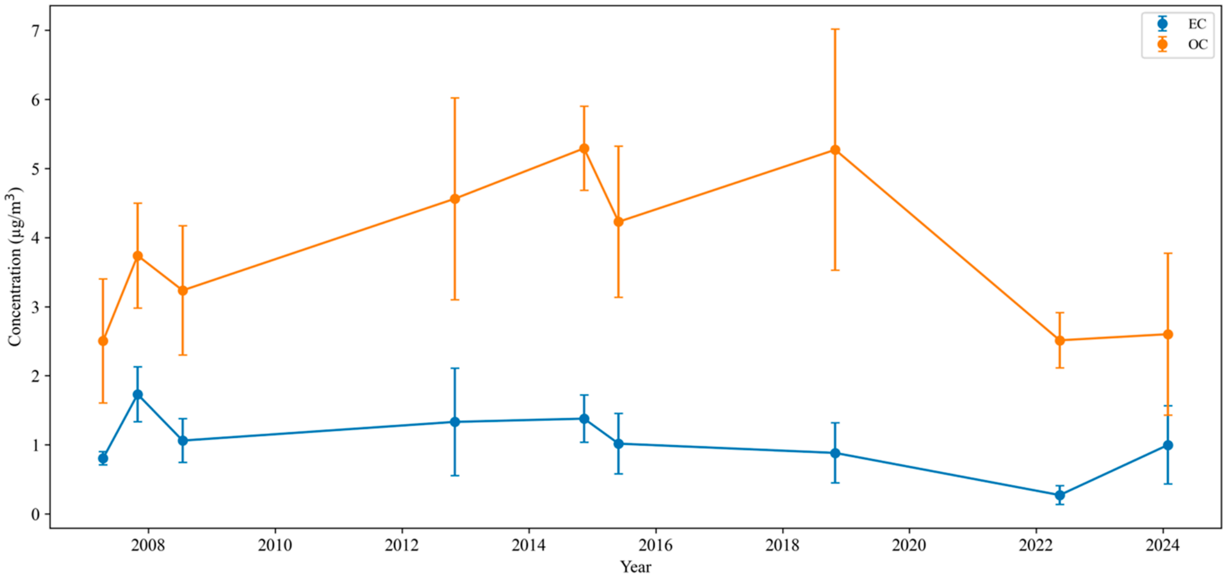

Figure S1. OC and EC concentrations in Los Angeles (2007–2024) on dates corresponding to DTT activity reported in prior studies.

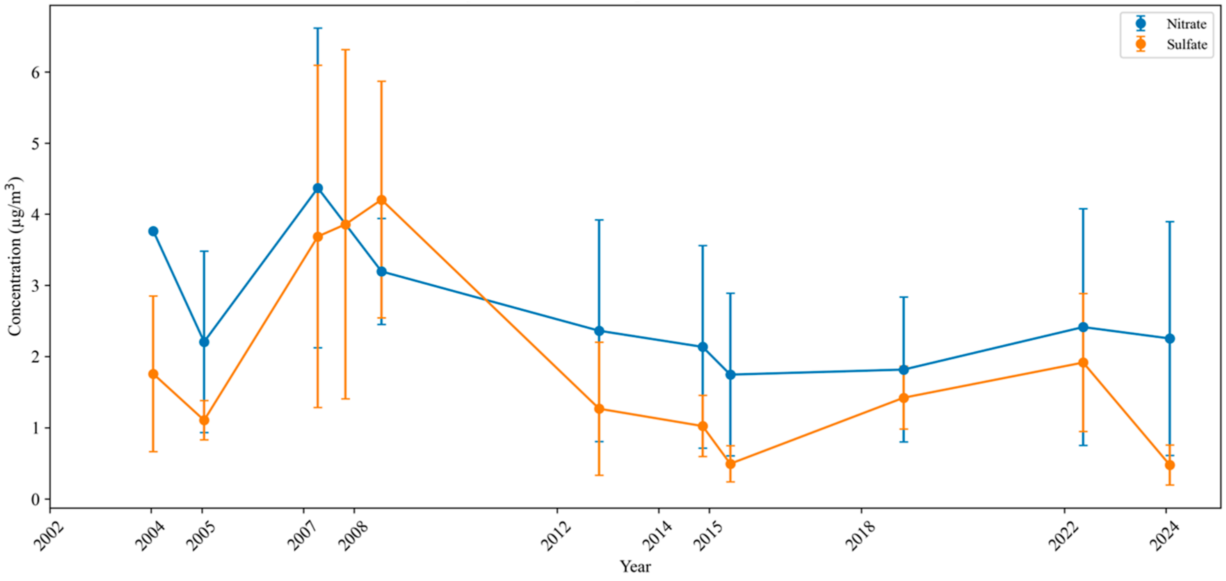

Figure S2. Sulfate and Nitrate concentrations in Los Angeles (2007–2024) on dates corresponding to DTT activity reported in prior studies.

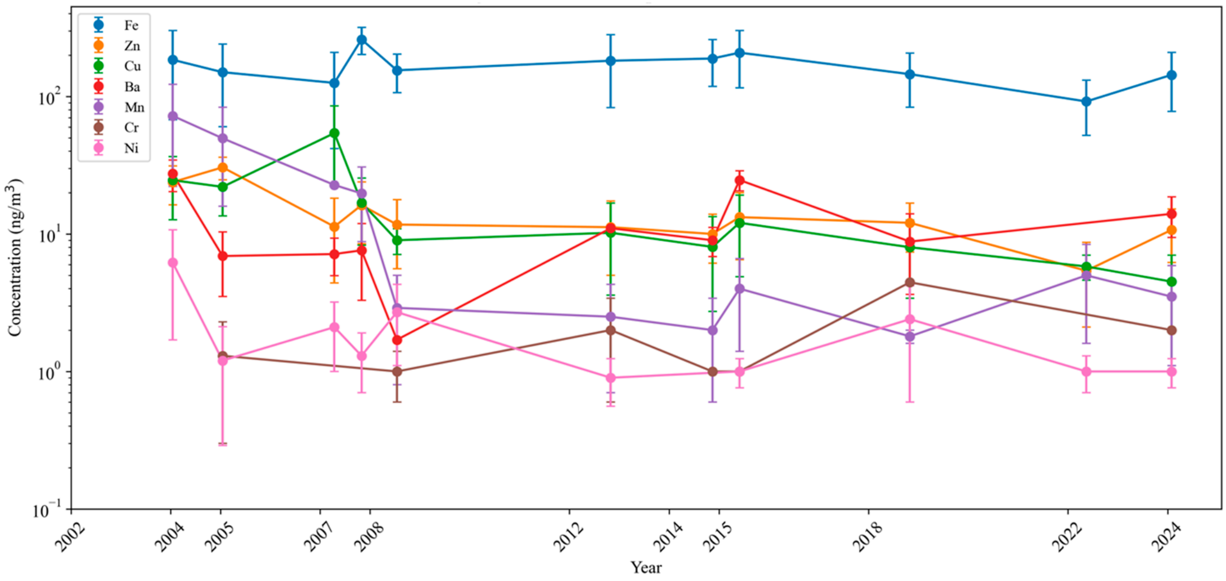

Figure S3. Metals' concentrations in Los Angeles (2007–2024) on dates corresponding to DTT activity reported in prior studies.

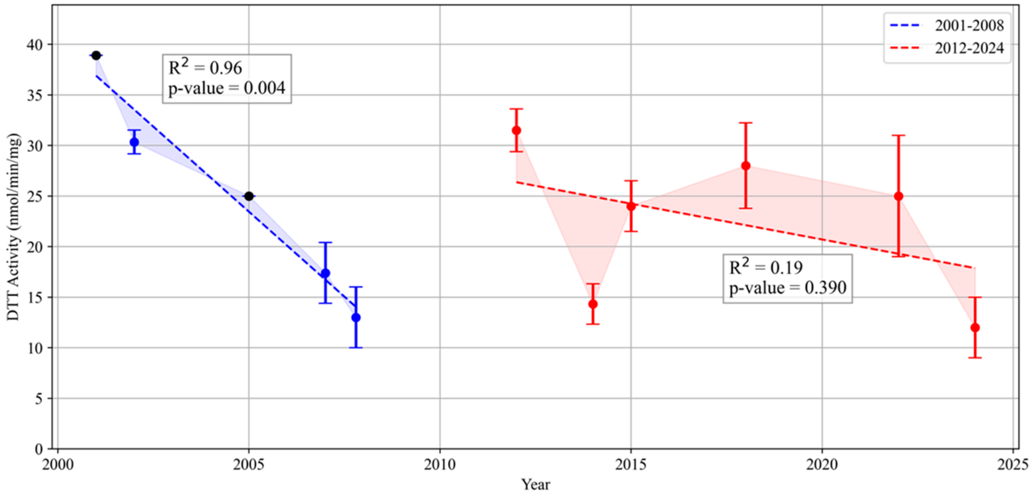

Figure S4. Long-term trends of DTT activity per mass of $PM_{2.5}$ in Los Angeles (2001–2024).
